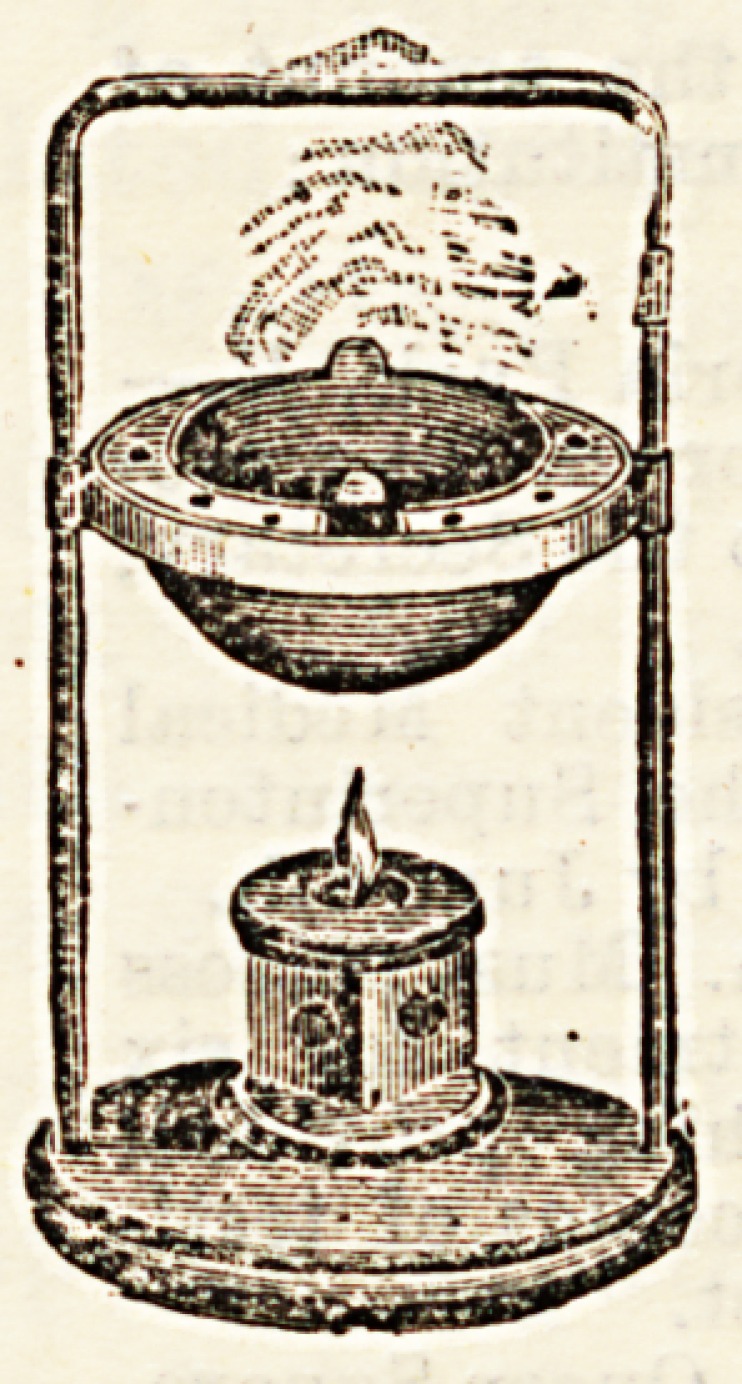# The Book World of Medicine and Science

**Published:** 1895-06-01

**Authors:** 


### THE BOOK WORLD OF MEDICINE AND SCIENCE.

A HANDBOOK OF HYGIENE. By A. M. DAVIES, D.P.H. (Camb.), late Assistant Professor of Hygiene, Army Medical School, Netley. (London: Charles Griffin and Co. 1895. Pp. 590.)

In its way this is really an admirable book. The author does not lay claim to originality. "To make himself acquainted, as far as he has been able, with what has been written on the subject by approved authorities, to compare and weigh different statements, to digest this information into as small a compass as seemed advisable," may not seem a high aim, but it is a most useful one, and in this case the object has been most thoroughly attained. The result is a most handy work of reference, full of information on the various points treated of. The subjects usually considered to pertain to hygiene are treated with considerable fulness, and a good deal of information is also given on various collateral topics. The article on "Food and Dieting" is very complete, and that on the "Causation and Prevention of Disease" is both interesting and well up to date. The subject of "Water and Water Supply" is carefully dealt with, but we may feel inclined to question the practical utility of the details given as to the biological examination, a thing which would hardly be undertaken without such a bacteriological training as would render them unnecessary. Some will be relieved to find no references made to sanitary law. Certainly the absence of abstracts of Acts of Parliament and of details regarding the special duties of medical officers of health in England gives the work a more scientific and cosmopolitan air; at the same time it is an omission which renders it less fitted than it otherwise would be for the use of English students.

MENTALLY ENFEEBLED CHILDREN. By FLETCHER BEACH, M.B., F.R.C.P.Lond., Physician to the West End Hospital for Nervous Diseases, Honorary General Secretary Medico-Psychological Association of Great Britain and Ireland, &c. (London: J. and A. Churchill. 1895. Price 1s. 6d.)

Dr. Fletcher Beach has condensed into thirty widely-printed pages a great deal of valuable matter concerning the mentally enfeebled. The variations of disease make it difficult to lay down any absolute rule of treatment, but for the average all the varieties of kindergarten work are useful. Paper-plaiting, sloyd-work, fret-work, and the like develop the intelligence and train the hands in co-ordination, while shapes and dimensions may be learned from such pastimes as brick-building. Progress, however, must always be slow, for the sensations as well as the intelligence of such children are dull, and endless experiments in touch, taste, and smell will be necessary to impress on them such knowledge of the different qualities of things as most children acquire unconsciously. Moreover, the brightest children are often the least satisfactory pupils, as in them an intelligence more nearly approaching the normal is balanced by a more volatile temper and a more capricious will. To such children it is difficult to impart moral ideas, while their very incapacity for being moved by the ordinary prudential considerations which restrain the sane from crime and violence, make it more necessary that the notions of duty towards their neighbour and responsibility to God should be implanted in them. Dr. Beach dwells on the importance of proper food, especially in the case of epileptics, and on the advantages of an institution, with the constant supervision of a physician and the instruction of trained teachers, over the homes of either rich or poor. He is sceptical as to the value of craniectomy in developing the intelligence of imbeciles, and trusts more to education than to any operation. On the other hand, he thinks that the administration of some preparation of the thyroid gland is useful in cases of a cretinoid variety. This little book is admirably concise, reasonable, and practical, and should be of use to all who have to deal with the mentally weak.

A NEW METHOD OF INHALATION. By W. H. SPENCER, M.D.Cantab., M.R.C.P. Pp. 54, illustrated. (London: The Scientific Press, 1895, 8vo.)

Dr. W. H. Spencer has had an unusually large experience in the treatment of medical diseases, and has for many years devoted his special attention to the treatment of tubercular and other affections of the lungs; we therefore welcome this concise and explicit statement of the results obtained by the method of treatment he has elaborated. There is a great deal of practical and scientific information conveyed in the narrow limits of this little book, and practitioners will do well to acquaint themselves with this method of inhalation. An automatic and continuous vaporiser has been designed by the author. It is stated to be the practical outcome of many carefully-conducted experiments, and seems a most efficient arrangement.

HOLIDAY TRAMPS THROUGH PICTURESQUE ENGLAND AND WALES. By H. H. WARNER. (Iliffe and Son.)

HOLIDAY TRAMPS is a lively little book, containing material for nine tours, each of about the length of a proverbial "parson's week" in various parts of England and Wales. The localities are well chosen with a view to giving an impression of various phases of scenery, from the peaceful prettiness of the Isle of Wight, Pett Level, and the New Forest, to the bolder beauties of Dartmoor and the Lakes. Excellent maps are given of each district, with tables of suggested walks and their distances from the larger towns. The wayside notes on botany and natural history make pleasant reading, and the geological hints are not too profound to be service to the holiday maker. It is a work likely to help many people to enjoy their summer in a healthy and restful manner in their own island, and for the American or Colonial, anxious to use his time to the best advantage while in England, it should prove a real boon, though such an one will be likely to resent the omission of Stratford-on-Avon and its neighbouring attractions from the itinerary.

## Figures and Tables

**Figure f1:**